# Use of high-flow nasal cannula as primary support for acute viral bronchiolitis

**DOI:** 10.62675/2965-2774.20260038

**Published:** 2026-01-14

**Authors:** Cássio Daniel Araújo da Silva, Roberta Botelho Monteiro, Larissa dos Santos Guarany, Rebeca Ferreira Costa, Guilherme Cherene Barros de Souza, Ana Paula Fernandes Moreira, Paula Cristina dos Santos Cabral, Ana Carolina Cabral Pinheiro Scarlato, Maria Fernanda de Andrade Melo e Araújo Motta, Patrícia Vieira Fernandes, Daniella Campelo Batalha Cox Moore, Saint Clair dos Santos Gomes, Fernanda Lima Setta

**Affiliations:** 1 Hospital Rios D’Or Rede D’Or São Luiz Rio de Janeiro RJ Brazil Hospital Rios D’Or, Rede D’Or São Luiz - Rio de Janeiro (RJ), Brazil.; 2 Hospital Unimed Rio de Janeiro RJ Brazil Hospital Unimed - Rio de Janeiro (RJ), Brazil.; 3 Fundação Oswaldo Cruz Instituto Fernandes Figueira Rio de Janeiro RJ Brazil Instituto Fernandes Figueira, Fundação Oswaldo Cruz - Rio de Janeiro (RJ), Brazil.

## INTRODUCTION

Acute viral bronchiolitis (AVB) is the infection of the lower airways, most common in the pediatric population, primarily caused by the respiratory syncytial virus (RSV). It typically follows a self-limited course with variable severity.^(1,2)^ Among respiratory support options, the high-flow nasal cannula (HFNC) has been promising for this population in several studies.^(1,3–5)^ However, only one Brazilian study has investigated this topic to date.^(6)^ Therefore, we aimed to describe the use of HFNC in a population with AVB, evaluating outcomes and success rates.

## METHODS

**Figure 1:**
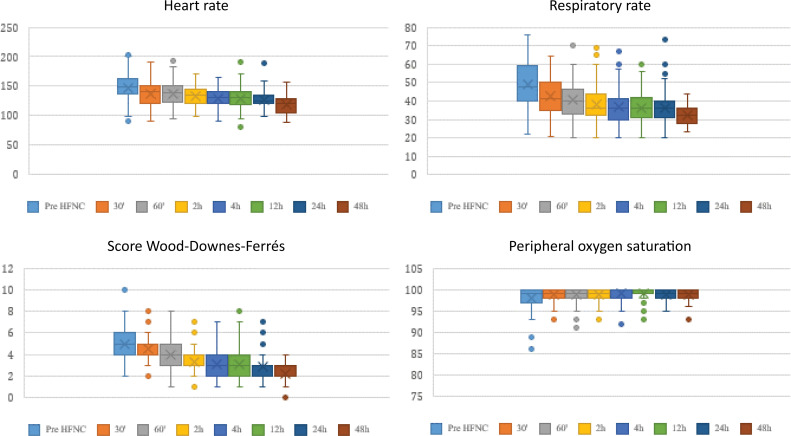
Vital signs evaluation before and after high flow nasal cannula initiation.

This retrospective study was approved by the local Ethics Committee (N°6.067.468) and conducted at a tertiary private hospital in Rio de Janeiro (RJ). All infants with AVB who received HFNC as their primary respiratory support during 30 months were selected, as per the sample selection flowchart provided in figure 1S (Supplementary Material).

The primary indication for HFNC was a "moderate" score (4 - 7 points) on the Wood-Downes-Ferrés scale,^(7)^ with a maximum flow rate of 2L/kg, as outlined in the protocol described in figure 2S (Supplementary Material). Demographic, clinical, and outcome data were recorded, along with reevaluation of vital signs before and after HFNC installation. Therapy was considered successful when HFNC did not require replacement with noninvasive ventilation (NIV) as rescue therapy. The data were analyzed in subgroups based on the success or failure of HFNC (using unpaired Student's t-test and Mann-Whitney tests, according to Bartlett's previous normality tests) and described as median values with their corresponding interquartile ranges. Excluded cases were not considered for statistical analysis.

## RESULTS

One hundred fifty-one HFNC cases were included in the study; 57% of these were male, with a median age of 6 months, RSV incidence of 35%, and pneumonia (23%) and viral co-infection (15%). The HFNC success rate was 75%. When stratifying cases by success or failure, we observed significant differences in the Wood-Downes-Ferrés variables at admission and at hospitalization (both higher in the HFNC Failure Group). The characterization of the study population is described in detail in table 1.

**Table 1:** Demographic profile and clinical characterization of the study population stratified into high flow nasal cannula success/failure groups

n (%)	Total	Success	Failure	p-value
	n = 151	n = 113 (75)	n = 38 (25)	
Male/Female (%)		M: 58.5 F: 41.5	M: 52.5 F: 47.5	0.471
Age (months)	6 (0 - 24)	7 (0 - 24)	6 (0 - 24)	0.192
Weight (kg)	8 (3 - 13.3)	8 (3 - 13.3)	7.2 (3.4 - 13)	0.077
RSV	53 (35)	35 (30.9)	18 (47.3)	0.056
Pneumonia	37 (24.5)	23 (20.3)	14 (12.3)	0.429
Co-infection	23 (15.2)	15 (13.2)	7 (6.1)	0.340
Wood-Downes scale at admission	5 (2 - 10)	5 (2 - 8)	5 (3 - 10)	0.035
Days of disease progression at admission	4 (1 - 18)	4 (1 - 18)	3 (1 - 15)	
Days of hospitalization	8 (1 - 38)	7 (1 - 38)	12 (6 - 35)	0.000
Days in use of HFNC	4 (1 - 24)	4 (1 - 12)	1 (1 - 15)	0.000

RSV - respiratory syncytial virus; HFNC - high flow nasal cannula. Results expressed as n. n (%) or median (interquartile range).

The median fraction of inspired oxygen (FiO_2_) at HFNC admission was 30%, which decreased to 25% within 48 hours. All vital signs and the Wood-Downes-Ferrés score progressively improved within the first half-hour of therapy, as shown in figure 1.

HFNC - high flow nasal cannula.

Vital signs before and after installation of the support. Error bars on graphs represent the interquartile range.

Noninvasive ventilation was used intermittently (with a daily limit of 6 hours) in 6% of cases, while 25% experienced HFNC failure and required rescue NIV. Of these, only ten infants experienced NIV failure due to deteriorated respiratory pattern or hemodynamic instability. Thus, the intubation rate in the study was 6%. There were no HFNC-related adverse event reports, and the mortality rate in the population was 0,6%.

## DISCUSSION

The clinical profile included young infants, predominantly male, but with a lower incidence of RSV than reported in the literature, around 60%.^(1–4)^ The stabilization of vital signs following HFNC initiation supports a possible positive response in the first hours of therapy, as observed.^(3,8)^ On the other hand, according to some authors, the clinical response to HFNC in AVB can be unspecified due to disease heterogeneity, highlighting the importance of well-defined criteria for therapy management.^(3,6,8)^

The frequent occurrence of SpO_2_ levels above the target value (96%) per the protocol indicates that weaning could have been encouraged, thereby avoiding the risks of hyperoxia and oxygen costs. According to Betters et al., infants are the population that primarily benefits from the mechanisms proposed by HFNC, probably because most are nasal breathers and have smaller airways than adults, which HFNC can progressively reduce the ventilator workload.^(8)^

The Brazilian study by César et al. made an interesting comparison between HFNC and nasal continuous positive airway pressure in moderate-to-severe AVB cases, noting that, in general, the modalities had similar treatment failure rates.^(6)^ We also noticed similar lengths of stay at the ICU and hospital, highlighting that the HFNC therapy is relatively new in many Brazilian services, which may imply difficulties in its understanding and management. Finally, we emphasize the importance of using protocols based on objective criteria for therapy, which enables precise indications and uniform management across all assistant care teams.

Limitations include a single-center study design with a population restricted to a private unit, which limits generalizability; therefore, further studies are needed to corroborate the findings presented here and compare the therapy with other forms of ventilatory support.

## Data Availability

The contents underlying the research text are included in the manuscript.
