# Supplementary material for: Use of high-flow nasal cannula as primary support for acute viral bronchiolitis
Source: Crit Care Sci. 2026 Jan 14;38:e20260038. doi: 10.62675/2965-2774.20260038 (PMC12977205; doi:10.62675/2965-2774.20260038)
Supplement: Supplementary file 1 [file 2965-2774-ccsci-38-e20260038-suppl1.pdf]

Critical Care Science
https://doi.org/10.62675/2965-2774.20260038

SUPPLEMENTARY MATERIAL

Use of high-flow nasal cannula as primary support for acute viral bronchiolitis

Cássio Daniel Araújo da Silva[1], Roberta Botelho Monteiro[1], Larissa dos Santos Guarany[1], Rebeca Ferreira Costa[1], Guilherme Cherene Barros de Souza[2], Ana Paula Fernandes Moreira[1], Paula Cristina dos Santos Cabral[1], Ana Carolina Cabral Pinheiro Scarlato[1], Maria Fernanda de Andrade Melo e Araújo Motta[1], Patrícia Vieira Fernandes[1], Daniella Campelo Batalha Cox Moore[3], Saint Clair dos Santos Gomes Junior[3], Fernanda Lima Setta[3]

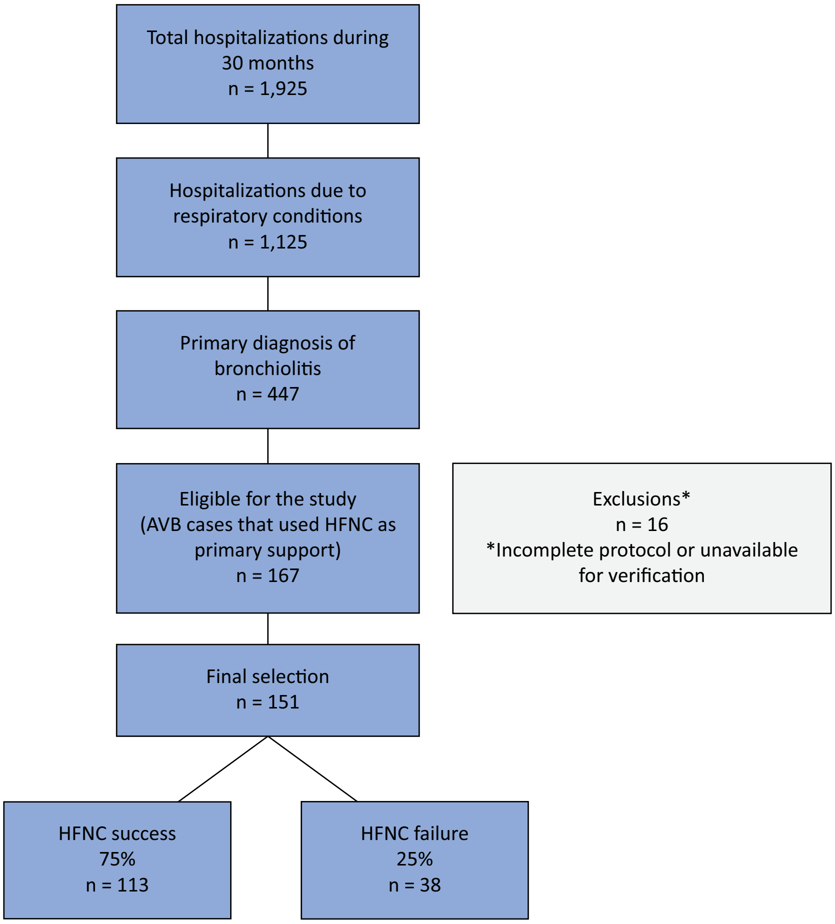

AVB - acute viral bronchiolitis; HFNC - high flow nasal cannula.
Figure 1S - Study population selection stages.

This is an open access article under the CC BY license (https://creativecommons.org/licenses/by/4.0/).

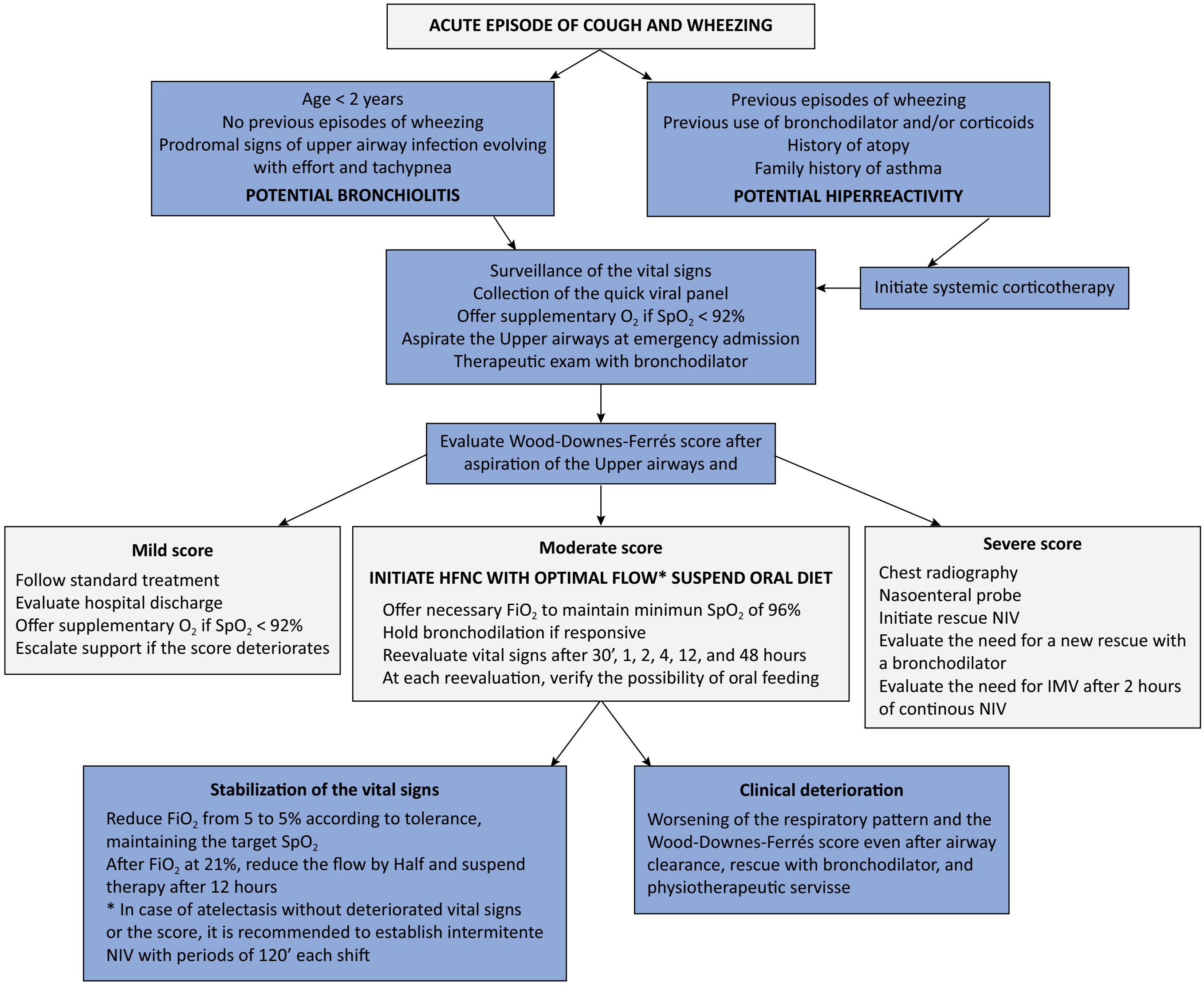

*The ideal HFNC flow rate will be calculated based on weight and can reach a maximum of 2.0L/kg.
- Up to 10kg: use 2L/kg.
- Over 10kg: use 2L/kg for the first 10kg and add 0.5L/kg.

Ex: child weighing 18kg: flow rate = 24L.

	0	1	2	3
Wheezing	None	End expiration	Entire expiratory phase	Inspiration and expiration
Retractions	None	Subcostal or lower intercostal	1 + supraclavicular + nasal flaring	2 + suprastemal + lower intercostal
Respiratory rate—breaths/min	< 30	31–45	46–60	>60
Heart rate—beats/min	< 120	> 120		
Inspiratory breath sounds	Normal	Regular, symmetrical	Markedly silent, symmetrical	Silent thorax, no wheezing
Cyanosis	Not present	Present		

A score of 1–3 points denotes mild bronchiolitis; 4–7 moderate bronchiolitis; and 8–14 ssevere bronchiolitis.

O_2 - oxygen; SpO_2 - peripheral oxygen saturation; FiO_2 - fraction of inspired oxygen; NIV - noninvasive ventilation; HFNC - high flow nasal cannula.

Figure 2S - Institutional protocol for the use of high flow nasal cannula in bronchiolitis.

Source: Adapted from: Flores-González JC, Matamala-Morillo MA, Rodríguez-Campoy P, Pérez-Guerrero JJ, Serrano-Moyano B, Comino-Vazquez P, et al.; Bronchiolitis of Cadiz Study group (BronCaS). Epinephrine improves the efficacy of nebulized hypertonic saline in moderate bronchiolitis: a randomized clinical trial. PLoS One. 2015; 10(11):e0142847. https://doi.org/10.1371/journal.pone.0142847
